# A community-based study to assess the prevalence and predictors of stunting among under-five children in Sheger City, Oromia, Ethiopia

**DOI:** 10.3389/fnut.2025.1479732

**Published:** 2025-01-28

**Authors:** Firomsa Botorie, Bilisom Balcha Abera, Abera Botorie, Asonya Abera, Abebe Dukessa Dubiwak, Tesfaye Getachew Charkos, Godana Arero Dassie

**Affiliations:** ^1^Lega Tafo Lega Dadi Health Centre, Sheger City, Ethiopia; ^2^School of Medicine, Faculty of Medical Sciences, Institute of Health, Jimma University, Jimma, Ethiopia; ^3^Department of Health Service Management and Police, Faculty of Public Health, Institute of Health, Jimma University, Jimma, Ethiopia; ^4^Oromia Regional Health Bureau, Addis Ababa, Ethiopia; ^5^College of Medicine and Health Sciences, Arbaminch University, Arbaminch, Ethiopia; ^6^Department of Epidemiology, Faculty of Public Health, Institute of Health, Jimma University, Jimma, Ethiopia; ^7^Department of Biomedical Sciences, Faculty of Medical Sciences, Institute of Health, Jimma University, Jimma, Ethiopia; ^8^School of Public Health, Adama Hospital Medical College, Adama, Ethiopia

**Keywords:** stunting, children, predictors, Sheger, under-five, Ethiopia

## Abstract

**Background:**

Stunting is a serious public health issue in Ethiopia. However, due to the scarcity of studies, little is known about the prevalence and predictors of stunting among children under the age of five in urban areas, especially those close to the capital city of the country (Addis Ababa). Thus, the aim of this study was to assess the prevalence of stunting and its predictors among children under the age of five in Lega Tafo Lega Dadi, Sheger City, Oromia, Ethiopia.

**Methods:**

A community-based cross-sectional study was conducted on 566 children under the age of five using a systematic random sampling technique. Data were collected through face-to-face interviews, structured questionnaires, and anthropometric measurements. Afterward, the data were entered into EpiData (version 4.7) and exported to SPSS 26 for analysis. Anthropometric indices were calculated using WHO Anthro software (version 3.2.2). Bivariate and multivariable logistic regression analyses were performed to identify candidate variables and associated factors, respectively. An adjusted odds ratio (AOR) and its 95% confidence interval (CI) were used to assess the strength and significance of the association. A *p*-value of <0.05 was considered statistically significant. The goodness-of-fit for the model was assessed using the Hosmer-Lemeshow test.

**Results:**

The prevalence of stunting was 18.9% (95% CI: 16, 22%) among under-five children in our study setting. Children whose mothers were daily laborers (AOR: 10.3), whose mothers’ education level was primary school (AOR: 4.3), whose fathers were daily laborers (AOR: 4), who were born into families with an average birth interval of ≤24 months (AOR: 7.9), who were from families with a size ≥5 (AOR: 7.3), who had a history of diarrhea (AOR: 6.3), who had meals ≤3 times per day (AOR: 13.9), who were underweight (AOR: 2.8), who were breastfed for less than 2 years (AOR: 5.6), who had low dietary diversity (AOR: 6.3), and who experienced food insecurity (AOR: 3.6) were identified as the predictors of stunting in under-five children.

**Conclusion:**

Approximately one-fifth of the under-five children were stunted in the study setting. Family occupational and educational status, average birth interval, family size, a history of diarrhea, meal frequency per day, underweight status, duration of breastfeeding, inadequate dietary diversity, and household food insecurity were all associated with stunting among the under-five children in the study setting.

## Introduction

Stunting is a condition that affects children’s nutritional status when their body length or height-for-age z-score is less than minus two standard deviations (SDs) compared to the standard population (1). Stunting often begins in utero, and the first 1,000 days of a baby’s life are a crucial period during which a child can become stunted. In developing countries, the majority of cases of stunting occurs during the first 2 years of life (2, 3). Most studies conducted in low- and middle-income countries have shown that growth faltering typically begins during pregnancy and by 2 years of age (4).

It is a severe and multigenerational growth impairment. Globally, 149 million (21.9%) children under the age of five were reported to be stunted, with more than half of them living in Asia (5, 6). Stunting is a major public health issue and a well-established indicator of chronic malnutrition that accurately reflects prior nutritional history and current environmental and socioeconomic conditions (6). Stunted children are more susceptible to disease and are at a higher risk of developing degenerative diseases in adulthood. Stunting not only affects health but can also impact the level of intelligence in children (7). Stunting during childhood impacts functional ability and causes growth delays, leading to short stature in adolescence and adulthood (8).

The prevalence of stunting in Africa is among the highest globally, and current trends indicate a stagnation rather than progress in addressing this issue (9). Stunting is a serious public health issue in Ethiopia (10). According to the Ethiopia Demographic and Health Survey (EDHS) 2019, the prevalence of stunting among children is 37% in Ethiopia and 35.6% in Oromia (11).

The WHO’s global target is to reduce childhood stunting by 40% in 2025 (12), and the reduction of stunting in children is also included in the United Nations Sustainable Development Goals. Therefore, it is recommended to establish policies aimed at preventing and reducing the incidence of stunting among children. However, these efforts are still not functioning effectively in the majority of low- and middle-income countries, including Ethiopia (13). Although several research studies have been conducted in Ethiopia, the majority of these studies have focused on rural areas rather than urban areas (14). As a result, little is known about the prevalence and predictors of stunting among children under the age of five in urban areas, especially those close to the capital city of the country (Addis Ababa). Sheger City is located very close to the boundary of the capital city of Ethiopia; however, no studies have been conducted in this area. Additionally, the majority of stunting-related studies have focused on children over the age of five; therefore, there is no sufficient evidence on stunting and its predictors among children under the age of five. Community-level predictors, in particular, have received little attention in studies on stunting in under-five children (15).

Assessing the prevalence of stunting and its predictors among under-five children at the community level is of paramount importance to identify high-risk cases and formulate appropriate intervention measures. Therefore, this study aimed to assess the prevalence of stunting and its predictors in Lega Tafo Lega Dadi, Sheger City, Oromia, Ethiopia. Hence, this evidence is crucial for policymakers and may aid public health planners in developing strategies for addressing the issue and enhancing children’s nutritional status.

## Methods and study participants

### Study design, area, and period

A community-based cross-sectional study was conducted in Lega Tafo Lega Dadi, Sheger City, Oromia, Ethiopia, which is located 25 km from Addis Ababa. The sub-city has four urban kebeles, two health centers, and 21 private health facilities. The total number of under-five children in the sub-city was reported to be 14,045 (16). The study was conducted from 1 March 2023 to 30 March 2023.

### Sample size and sampling procedure

**Figure 1:**
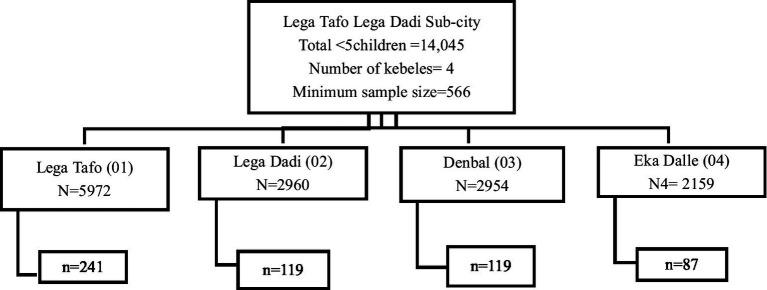
Schematic presentation of sampling procedure of under-five children in Lega Tafo Lega Dadi Sub-city. N = total under five children in the kebele, n = sample size of under five children in the kebele.

A single population proportion formula was used to calculate the sample size (17), assuming a 95% confidence level, a 3% margin of error, and a 14% proportion of stunting based on a previous study (11). After adjusting for a 10% non-response rate, the final sample size was 566. These 566 under-five children were proportionally allocated among four kebeles in Lega Tafo Lega Dadi (Figure 1). The systematic random sampling method was used to select households, using the interval that was determined for each kebele. The first household was selected using simple random sampling. When more than one eligible child was available in the selected household, simple random sampling was used to select one participant.

### Eligibility criteria

Under-five children who had been living in the study area for more than 6 months were included in the study. However, children who were reported to be seriously ill or hospitalized, neonates (less than 4 weeks old), preterm infants, and those with congenital anomalies were excluded from the study.

### Variables in the study

Stunting was the dependent variable, while family size, income status, maternal and paternal education status, occupation, place of residence, the Household Food Insecurity Access Scale (HFIAS), dietary diversity, child’s age, sex, morbidity status, history of diarrhea, time of initiation of breastfeeding, duration of exclusive breastfeeding, total length of breastfeeding, time to start complementary feeding, food groups, methods of feeding, immunization status, height of the mother, maternal care/antenatal care (ANC) visits, birth interval, and place of delivery were the independent variables.

### Data collection procedure and instruments

Data were collected using structured questionnaires that were initially prepared in English, then translated into Afan Oromo and back into English to ensure consistency. The participants were then interviewed in the local language (Afan Oromo). Anthropometric measurement tools (a standiometer and an infantometer for height, and a weight scale for weight) were used to determine the height and weight of the children, according to the WHO standards. Four health extension workers and two health officers were data collectors and supervisors, respectively.

### Data quality assurance

Two days of training on data collection tools and procedures were provided to the data collectors and supervisors before the designated data collection date. The supervisors checked the questionnaire daily for completeness. A pre-test of the questionnaire was conducted on 5% of the respondents (28 participants) at Walgaho Kebele households to assess the accuracy of the responses and to estimate the time required.

### Data processing and analysis

Before the analyses, the data were checked for completeness and inconsistencies, and then entered into EpiData (version 4.7). Subsequently, the data were exported to SPSS 26 and then cleaned, categorized, coded, and analyzed. Frequencies and percentages were used to describe sociodemographic, maternal, and child-caring practices. Anthropometric indices were calculated using WHO Anthro software (version 3.2.2) for child growth standards. Before conducting a multivariable binary logistic regression analysis, collinearity was checked to assess the multicollinearity assumption using the variance inflation factor (VIF). Bivariate logistic regression was performed for each independent variable. In addition, univariate binary logistic regression was performed for each independent variable, and the variable with a *p*-value less than 0.25 was considered a candidate for the multivariable logistic regression. The backward Wald method was used to construct the final regression model. An adjusted odds ratio (AOR) and its 95% confidence interval (CI) were used to assess the strength and significance of the association. The independent variables with a *p*-value less than 0.05 in the multivariable logistic regression analysis were considered statistically significant. The goodness-of-fit for the model was assessed using the Hosmer-Lemeshow test.

## Results

### Sociodemographic characteristics of the under-five children in Lega Tafo Lega Dadi

A total of 566 under-five children from Lega Tafo Lega Dadi participated in this study. Of these, 296 (52.3%) were male participants, and more than half were under the age of 2 years, accounting for 334 participants (59.0%). The majority of the parents of the children reported being Orthodox religious followers, accounting for 65% of the participants. In addition, 32.7% of the mothers/caregivers had secondary school education, 462 (81.6%) of the parents of the study participants were married, and 27.7% of the mothers/caregivers reported being merchants (Table 1).

**Table 1:** Socio-demographic characteristics of under five children and their families in the Lega Tafo Lega Dadi, Sheger city, Oromia, Ethiopia, 2023.

Variables	Category	Frequency *N* (%)
Age of child (in month)	0–6	148 (26.1)
	6–11	58 (10.2)
	12–23	128 (22.6)
	24–36	108 (19.1)
	37–59	124 (21.9)
Sex of child	Male	296 (52.3)
	Female	270 (47.7)
Head of the household	Male	462 (81.6)
	Female	104 (18.4)
Residence area	Urban	543 (95.9)
	Rural	23 (4.1)
Marital status parent/care giver	Married	462 (81.6)
	Others^*^	104 (18.4)
Religion of parent/care giver	Orthodox	368 (65)
	Muslim	119 (21)
	Protestant	76 (13.4)
	Others^┼^	3
Ethnicity of parent	Oromo	404 (71.4)
	Amhara	132 (23.3)
	Others^┼┼^	30 (5.3)
Educational status of mothers/care giver	Degree and above	84 (14.8)
	Elementary	131 (23.1)
	Secondary	185 (32.7)
	Diploma	135 (23.9)
	Others^**^	31 (5.5)
Educational status of father	Degree and above	134 (23.7)
	Elementary	133 (23.5)
	Secondary	154 (27.2)
	Diploma	94 (16.6)
	Others^**^	51 (9.0)
Occupation of mother/care giver	Government or NGO employee	145 (25.6)
	Housewife	141 (24.9)
	Merchant	157 (27.7)
	Daily Laborer	104 (18.4)
	Others^♀^	19 (3.4)
Occupation of father	Government or NGO employee	188 (33.2)
	Merchant	114 (20.1)
	Daily Laborer	188 (33.2)
	Others^♀^	76 (13.4)
Family size	≤4	379 (67.0)
	≥5	187 (33.0)
Family income	<1,000	31 (5.5)
	1,000–2,000	100 (17.7)
	≥2,000	435 (76.9)
Average birth interval	≤24 Months	192 (33.9)
	>24 Months	374 (66.1)

^*^Single, widow, ^**^illiterate, read and write, ^♀^Farmer, Waiter, ^┼^Catholic, ^┼┼^Gurage, Tigre.

### Child health status, dieting habits, and sanitation practices

More than half of the study participants had no illness/infection in the 2 weeks prior to the survey, and more than three-fourths of the under-five children had no history of diarrhea in the past year. Regarding breastfeeding, 549 (97.0%) were breastfed; of these, 520 (91.9%) of the under-five children were breastfed for more than 2 years (Table 2).

**Table 2:** Under-five child health status, feeding habits, and sanitation of study participants in Lega Tafo, Lega Dadi, Sheger City, Oromia, Ethiopia, 2023.

Variables	Category	Frequency *N* (%)
Illness/infection in the last 2 weeks prior to the survey	Yes	179 (31.6)
	No	387 (68.4)
Child history of diarrhea the last 1 year	Yes	117 (20.7)
	No	449 (79.6)
History of breast feeding	Yes	549 (97.0)
	No	17 (3.0)
Initiation of breast feeding after birth	Immediately (<1 h)	9 (1.6)
	1–24 h	457 (80.7)
	24––72 h	58 (10.2)
	≥72 h	25 (4.4)
Duration of exclusive breast feeding	<6 months	451 (79.7)
	≥6 months	115 (20.3)
Duration of total breast feeding	<2 years	46 (8.1)
	≥2 years	520 (91.9)
Main types/group of diet they did feed	Animal product	197 (34.8)
	Fruit	96 (17.0)
	Others^*^	273 (48.2)
Frequency of meal per day	≤3 times	187 (33.0)
	>3 times	379 (67.0)
Sources of drinking water	Protected	550 (97.2)
	Unprotected	16 (2.8)
Household food security status (HFIAS)	Secure	415 (73.3)
	Insecure	151 (26.7)
Child DDS score	Adequate	429 (75.6)
	Inadequate	137 (24.2)
Availability of latrine	Yes	550 (97.2)
	No	16 (2.8)
Type of latrine they use	Latrine with water	132 (23.3)
	Latrine without water	413 (73.0)
	Public latrine	5 (0.91)

*Vegetables, cereal, legumes.

### Prevalence of stunting in Lega Tafo Lega Dadi

**Figure 2:**
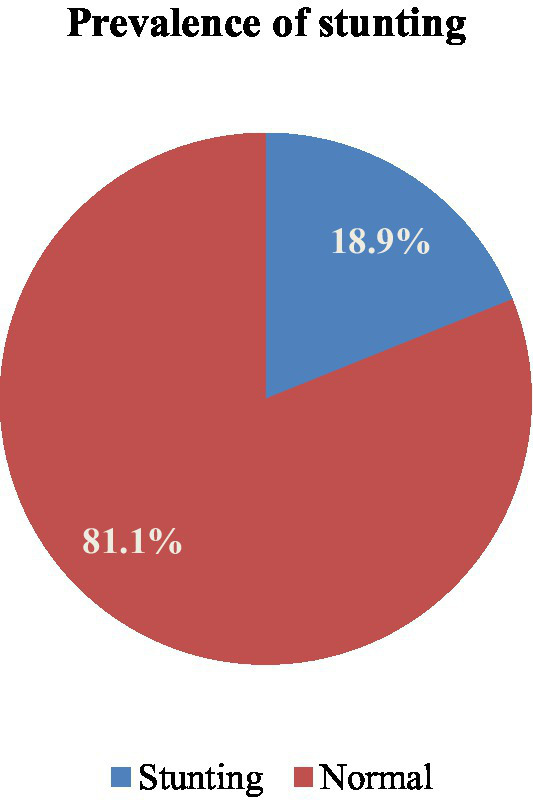
Prevalence of stunting among under-five children in Laga Tafo, Laga Dadi in 2023.

Of the 566 under-five children in our study population, 107 [18.9% (95% CI: 16, 22%)] were stunted (Figure 2).

### Factors associated with stunting among the under-five children in the study setting

In this study, the occupational status of the mothers had a statistically significant association with the stunted development of the children. The children born to mothers who were government or NGO employees were 6.36 times more likely to be stunted than those born to mothers who were housewives (AOR: 6.36; 95% CI: 1.67, 24.24). The odds of being stunted among the children whose mothers were merchants were 5.57 times higher than those whose mothers were housewives (AOR: 5.57; 95% CI: 1.51, 20.56). Likewise, the children born to mothers who were daily laborers were 10.3 times more likely to be stunted than those whose mothers were housewives (AOR: 10.30; 95% CI: 3.12, 34.03). Furthermore, the children born to mothers who had primary school education were 4.28 times more likely to be stunted than those born to mothers who had a degree or higher (AOR: 4.28; 95% CI: 1.01, 18.18). The under-five children whose fathers were daily laborers were 4 times more likely to be stunted than those whose fathers were government or NGO employees (AOR: 4; 95% CI: 1.25, 12.84). The children born into families with an average birth interval of ≤24 months were 7.99 times more likely to be stunted compared to those born into families with an average birth interval of >24 months (AOR: 7.99; 95% CI: 3.18, 20.07).

Moreover, the odds of being stunted among the children who had a history of diarrhea in the past year were 6.34 times higher compared to those who did not have a history of diarrhea in the past year (AOR: 6.34; 95% CI: 2.62, 15.35). The under-five children who were breastfed for less than 2 years had more than five times the likelihood of being stunted compared to those who were breastfed for 2 years or more (AOR: 5.6; 95% CI: 1.3, 23.3). The children whose families had five or more children were 7.26 times more likely to be stunted than those whose families had four or fewer children (AOR: 7.26, 95% CI: 4.56, 11.57).

The odds of stunting among the children who ate ≤3 times per day were 13.90 times higher compared to those who ate >3 times per day (AOR: 13.90; 95% CI: 4.68, 42.15). The odds of stunting were 2.75 times higher among the children who were underweight compared to the children with normal weight (OR: 2.75; 95% CI: 1.14, 6.60). The odds of stunting among the children whose households experienced food insecurity were 3.55 times higher compared to those whose households experienced food security (AOR: 3.55; 95% CI: 1.51, 8.38). The children who had inadequate dietary diversity were 6.26 times more likely to be stunted than those who had adequate dietary diversity (AOR: 6.26; 95% CI: 2.76, 14.17) (Table 3).

**Table 3:** Factors associated with stunting among under-five children (*n* = 566) in Lega Tafo, Lega Dadi Sub-city, Oromia, Ethiopia.

Variables	Category	Stunting		COR (95% CI)	AOR (95% CI)	*p*-value
		Yes	No			
Occupation of mother	Housewife	24	117	1	1	
	Government/NGO employee	13	132	0.48 (0.23, 0.99)^**^	6.36 (1.67, 24.24)	0.007
	Merchant	27	130	1.02 (0.55, 1.85)	5.57 (1.51, 20.56)	0.01
	Daily laborer	39	65	2.93 (1.62, 5.29)^** *^	10.30 (3.12, 34.03)	<0.001
Educational status of mothers/care takers	Degree and above	8	76	1	1	
	Primary	30	101	2.82 (1.23, 6.50)^*^	4.28 (1.01, 18.18)	0.049
Paternal occupational status	Government or NGO employee	13	171	1		
	Daily laborer	60	132	5.98 (3.15, 11.35)^***^	4.00 (1.25, 12.84)	0.02
Average birth interval	≤24 Months	64	128	3.85 (2.49, 5.96)^***^	7.99 (3.18, 20.07)	<0.001
	>24 Months	43	331	1	1	
Family size	≤4	32	347	1	1	
	≥5	75	112	15.35 (5.90, 39.91)^***^	7.26 (4.56, 11.57)	<0.001
Child’s has history of diarrhea in the last 1 year	Yes	55	62	6.77 (4.26, 10.77)^***^	6.34 (2.62, 15.35)	<0.001
	No	52	397	1	1	
Total duration of breast feeding	<2 years	2	44	11.68 (1.28, 86.73)^**^	5.57 (1.33, 23.33)	0.029
	≥2 years	105	415	1	1	
Meal frequency per day	≤3 times	80	107	9.75 (5.99, 15.86)^***^	13.90 (4.68, 42.15)	<0.001
	>3 times	27	352	1	1	
Food security status (HFIAS)	Secure	34	381	1	1	
	Insecure	73	78	10.49 (6.53, 16.85)^***^	3.55 (1.51, 8.38)	0.004
Underweight	No	37	388	1	1	
	Yes	70	71	10.34 (6.45, 16.57)^***^	2.75 (1.14, 6.60)	0.024
Diet diversity (DDS)	Adequate	39	390	1	1	
	Inadequate	68	69	9.86 (6.16, 15.76)^***^	6.26 (2.76, 14.17)	<0.001

^*^p < 0.25, ^**^p < 0.05, ^***^p < 0.001; AOR, Adjusted Odds Ratio; CI, Confidence Interval; COR, Crude Odds Ratio.

## Discussion

Overall, the prevalence of stunting in this study was 18.9%. The findings of this study are in agreement with those of a study conducted elsewhere in Ethiopia (19.7%) (18) and in Addis Ababa (18.5%) (19). The similarity implies that in developing countries, the nutritional status does not change over time and the pattern of stunting persists as children age. It could also be explained that as children grow older, their bodies’ need for nutrients increases without a corresponding increase in nutrient supply (20). In contrast to the current findings, studies conducted in different parts of Ethiopia, including the one conducted by the EDHS 2016 and in West Gojam Zone, Debre Berhan town, and Addis Ababa, reported higher prevalences of stunting among under-five children, with rates of 45.8, 43.2, 41.0, and 38.0%, respectively (15, 21–23). The discrepancy could be attributed to socio-demographic differences, variations in sample size, and differences in study periods. The nutritional status of under-five children is critical as it can make them more susceptible to disease and infection, hinder their mental and physical development, and increase the likelihood of stunting, preventing them from reaching their full height and cognitive potential compared to children with normal nutrition (24).

According to the present study, stunting is significantly associated with the mother’s occupation and educational status, paternal occupational status, family size, average birth interval, total duration of breastfeeding, history of diarrhea in the past year, meal frequency per day, the HFIAS, underweight status, and dietary diversity. Similar results were observed in studies conducted in Ethiopia, particularly in West Gojam (21), the West Guji Zone, Oromia (24), and northern Ethiopia (25).

Our findings reported that the under-five children whose mothers were daily laborer were more likely to be stunted than those whose mothers were housewives. A previous study from Bangladesh (26) corroborates our finding, suggesting that mothers involved in work, such as daily labor, may lack sufficient time to care for their children. This situation leads to poor hygiene practices, inadequate health-seeking behavior, and poor feeding practices, ultimately resulting in the child becoming undernourished (26). Likewise, the under-five children born to fathers who were daily laborers were 4 times more likely to be stunted than those whose fathers were government or NGO employees. Various studies conducted in different areas have shown that fathers play a significant role in the prevention of stunting (27, 28). Daily laborers may be linked to low household income, as a study conducted in Addis Ababa showed that low household income is one of the factors associated with household food insecurity (29), which is a well-known predictor of stunting.

In the current study, maternal educational status was statistically significantly associated with stunting among the under-five children. Specifically, the children whose mothers had primary education were more likely to be stunted than those whose mothers had a degree or higher education. This finding is supported by the findings of previous studies conducted in Ethiopia (30, 31), Nigeria (32), China, and Indonesia (33, 34). It is known that more literate mothers are generally more concerned about their child’s wellbeing than illiterate or less literate mothers (35). This may also be due to the fact that an educated mother is more likely to have better knowledge about the importance of dietary diversity and which foods provide greater nutritional value. Educated mothers may also have greater decision-making abilities and confidence at home and may be more effective in improving their family’s and children’s nutritional status.

The study result revealed that the children with a birth interval of <24 months were more likely to be stunted compared to the children with a birth interval of ≥24 months. This finding is supported by the findings of studies conducted in Ethiopia and Bangladesh (19, 36). When the birth interval is short, there is a higher probability of premature cessation of breastfeeding, which may lead to undernutrition, such as stunting. This study pointed out that stunting was strongly associated with family size. The children born into families with five or more children were more likely to experience stunting than those born into families with four or fewer children. This finding is in line with the findings of the studies conducted in Ethiopia (19, 25, 30, 31, 37). In addition, this finding is consistent with the findings of studies conducted in Vietnam, Bangladesh, Nigeria, and Ghana, where children from families with more than two children were more likely to be stunted than children from families with fewer than two children (32, 36, 38, 39). Food deficiencies are likely to increase as family size grows, leading to food insecurity, especially in families with a fixed income. A larger family size means less food is available for each family member compared to smaller families with the same economic resources. This may also be explained by the higher prevalence of infections or diseases in such families due to the overcrowded living conditions, which create a favorable environment for disease transmission from one family member to a child, potentially leading to childhood malnutrition (40).

Furthermore, the duration of breastfeeding was statistically significantly associated with the risk of stunting in the study. The children who were breastfed for less than 24 months were more likely to be stunted than the children who were breastfed for 24 months or more. This finding is supported by the finding of a previous study conducted in Ethiopia (41). A high prevalence of stunting was observed among the children with a history of diarrhea in the past year. This finding is in line with the finding of a study conducted in Ethiopia (21). Numerous studies have shown a reciprocal relationship between diarrhea and malnutrition: diarrhea leads to malnutrition, particularly undernutrition such as stunting, while malnutrition is also a predisposing factor for diarrhea. In our study, underweight was another predictor of stunting among the under-five children. Underweight is defined as low weight-for-age. A child who is underweight may be stunted, wasted, or both (42).

The under-five children living in food-insecure households were more likely to become stunted than their counterparts. This finding is in line with the findings of studies conducted in Nigeria and Ethiopia (32, 37). The possible reason for this is that the majority of the families in this study had low to medium socio-economic status, which are immediate causes of undernutrition. Low-income households are especially vulnerable to changes in food prices. In addition, food-insecure households do not have the ability to purchase diversified and nutritionally balanced food.

Finally, the present study showed that dietary diversity was statistically significantly associated with stunting. The children from families with inadequate dietary diversity were more likely to be stunted than children from families with adequate dietary diversity. Dietary diversity is an important component of children’s diet quality; consuming a wider variety of foods and food groups is linked to higher nutritional adequacy (43). A similar finding was reported in studies conducted in Iran and India (44). This can be explained by the fact that the lack of diversity is a particularly severe problem among poor populations in the developing world, where diets are predominantly based on starchy staples, with few or no animal products and only seasonal fruits and vegetables (45). Everyone needs a variety of foods to live a healthy life and meet basic nutritional requirements. A diverse diet provides essential micronutrients and adequate energy. The majority of the micronutrients we need come from our daily diet, so consuming a well-balanced diet rich in a variety of foods is essential. Given the wide range of health issues and the rising prevalence of diet-related chronic diseases, dietary diversity is crucial for preventing chronic diseases, including stunting in children (21).

## Conclusion

The prevalence of stunting among the under-five children in the current study area was 18.9%, which is considered moderate. In this study, stunting in the children under the age of five was predicted by maternal educational and occupational status, paternal occupational status, average birth interval, family size, history of diarrheal disease in the past year, duration of breastfeeding, daily diet frequency, the HFIAS, underweight status, and diet diversity. Therefore, various levels of government stakeholders and other concerned organizations should work to improve maternal educational status, enhance household food security, and empower vulnerable families to provide proper nutrition for their children and prevent diarrhea and related diseases caused by poor hygiene and sanitation.

## Data Availability

The original contributions presented in the study are included in the article/supplementary material, further inquiries can be directed to the corresponding author.
